# Complete Hydrothorax After Revision Shoulder Arthroscopic Surgery: A Case Report

**DOI:** 10.7759/cureus.23590

**Published:** 2022-03-28

**Authors:** Breanna Connett, Eric F Krohn, John Hilu

**Affiliations:** 1 Surgery, Beaumont Health, Dearborn, USA; 2 Orthopedics, Good Samaritan Regional Medical Center, Corvallis, USA; 3 Cardiothoracic Surgery, Beaumont Health, Dearborn, USA

**Keywords:** interscalene block, hydrothorax, postoperative complication, pleural effusion, shoulder arthroscopy

## Abstract

Shoulder arthroscopy has become very popular in recent years. While generally quite safe, it is subject to its own complications when compared to open surgery. Due to the high volume of fluid, postoperative pleural effusions are a known complication. Presented here is an extreme case of this with complete hydrothorax on the operative side requiring chest tube placement and admission.

## Introduction

Shoulder arthroscopy has greatly increased in popularity over the last few decades. One of the key advantages of arthroscopic surgery over open techniques is the lower reported complication rate [1]. While most complications are relatively mild, there are a few instances where complications can be devastating and if not identified and treated promptly, can lead to significant morbidity or even death. In this study, we present the case of a revision arthroscopic acromioclavicular ligament reconstruction complicated by a dangerous postoperative ipsilateral complete hydrothorax, which required tube thoracostomy to treat.

## Case presentation

A 64-year-old male with a weight of 96 kg, height 185.4 cm, calculated BMI of 27.92 underwent revision left shoulder arthroscopic acromioclavicular (AC) ligament reconstruction. The patient’s prior medical history included hypertension, coronary artery disease with stent placement, and atrial fibrillation with rapid ventricular response. His home medication included only aspirin. He underwent his initial operation five months prior without significant complication. Postoperatively, he had recurrent separation of the AC joint at six months. At his preoperative workup, he was found to have no pulmonary system complaints or concerning findings on examination.

Prior to the operation, the patient was given a single-shot interscalene block under ultrasound guidance. Then he was placed under general anesthesia, intubated, and positioned in the beach chair position. The arm was controlled with the Trimano (Naples, FL: Arthrex Inc.) arm holder. The case proceeded with the placement of the posterior, then anterior portals. Debridement of labral tissues and scar was undertaken before moving to the coracoid. The undersurface of the coracoid was debrided and then sharp dissection was carried down to the coracoid. The old reconstruction was removed and the prior drill hole through the coracoid was still intact and so no repeat drilling was undertaken. Using the AC Dog Bone Button and Fibertape (Naples, FL: Arthrex Inc.) along with posterior tibial tendon allograft, the acromioclavicular ligaments were reconstructed. The wounds were closed and there were no reported complications. The patient was extubated and transferred out of the operative room without issue. The procedure time from incision to extubation was noted to be three hours.

**Figure 1:**
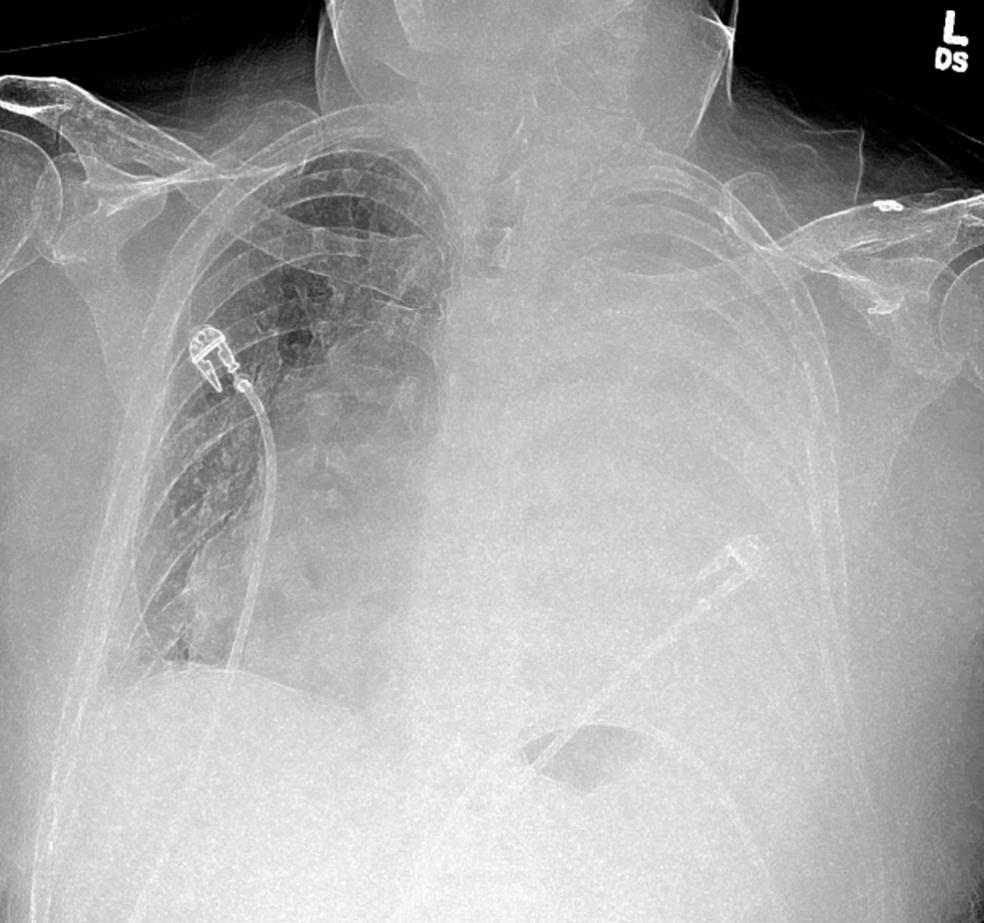
Initial chest x-ray of the patient

**Figure 2:**
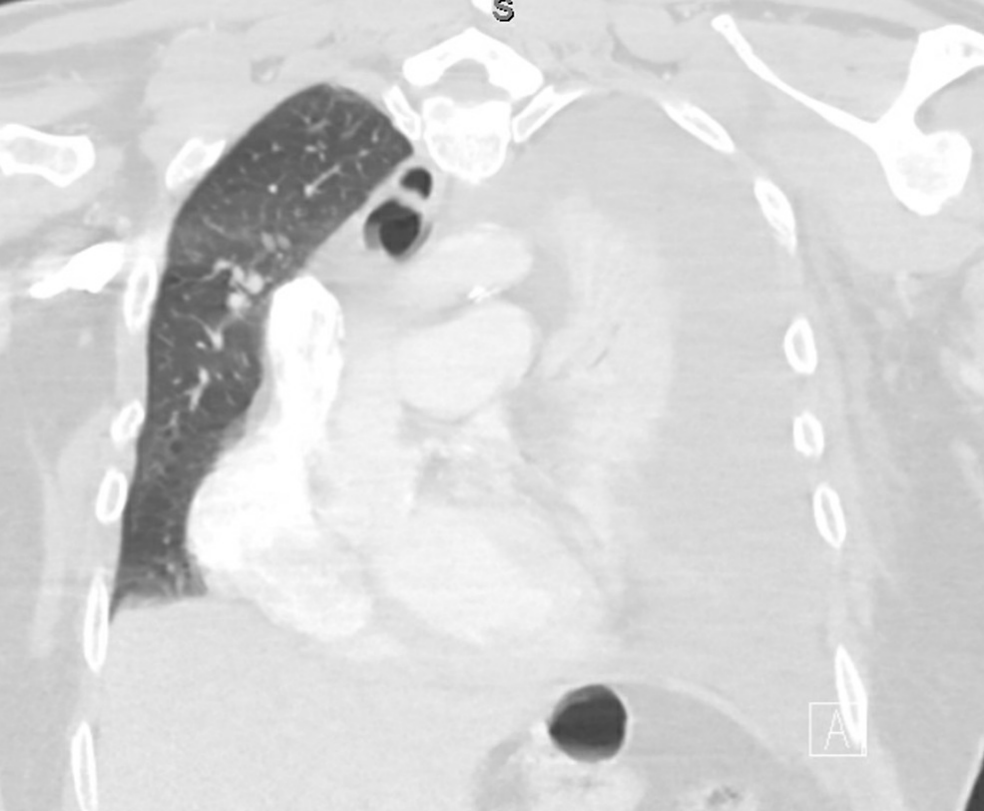
Coronal CT with evidence of large left hydrothorax with mediastinal shift

Within 60 minutes of transfer to the postoperative anesthesia care unit (PACU), the patient started to develop chest pain and shortness of breath. He was initially hypoxic with SpO_2_ at 89%, but 3 L O_2_ by nasal cannula improved his oxygenation to above 92%. Apart from the hypoxia and his subjective complaints, his vital signs were stable. An EKG was performed which showed no concerning changes. His troponin level was also checked, but was within normal limits. Urgent chest x-ray was performed which revealed complete opacification of left hemithorax with mediastinal shift consistent with complete hydrothorax (Figures 1, 2).

**Figure 3:**
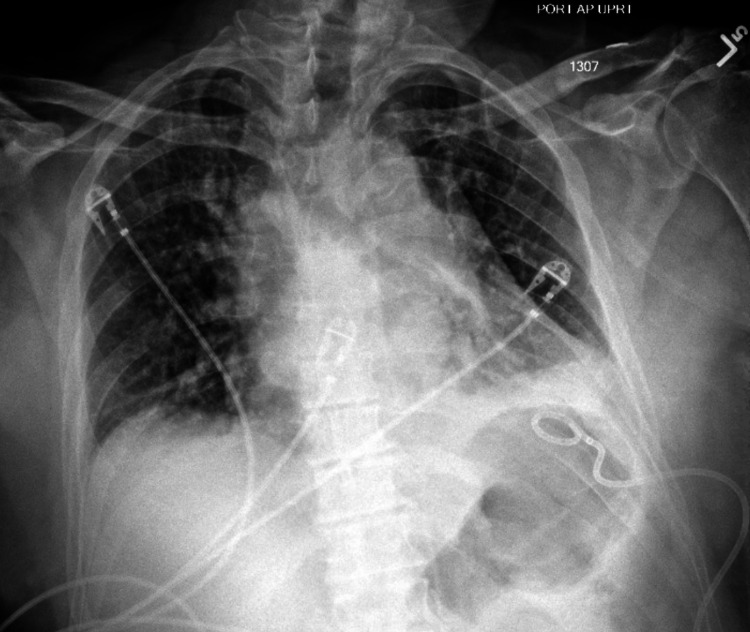
Chest x-ray shows post pigtail catheter placement

**Figure 4:**
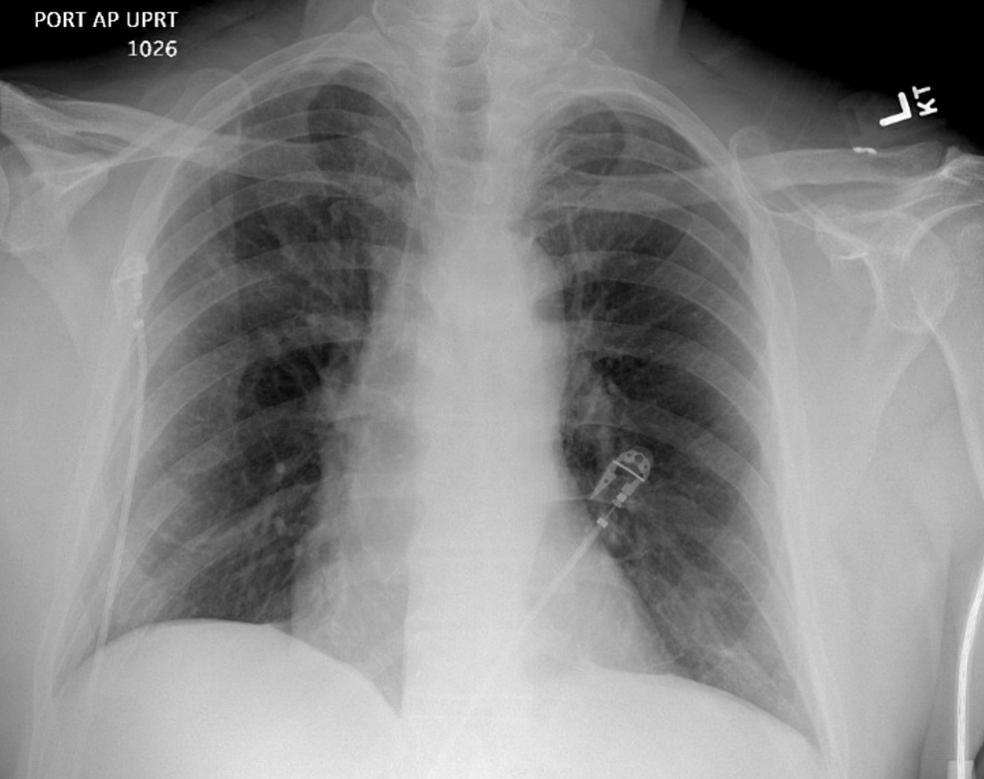
Chest x-ray after removal of pigtail catheter

He then underwent image-guided left chest tube placement with the department of interventional radiology with an initial output of 1000 mL blood-tinged fluid and placement of a 10.2 Fr chest tube (Figure 3). Over the next day, there was an additional 900 mL of output from the chest tube. It was removed on the first postoperative day after x-rays demonstrated complete resolution of the hydrothorax (Figure 4). He was discharged on postoperative day four.

## Discussion

Fluid extravasation after shoulder arthroscopy has been well reported in the literature since 1990 with reports of edema of the chest wall, neck, and face as well as airway compression and respiratory compromise [2-6]. A literature review on fluid extravasation into the pleural space after shoulder arthroscopy resulted in only one other patient with a large enough pleural effusion that required drainage postoperatively [6]. Most of the time, the pleural effusions are small volumes and not clinically significant.

Pleural effusions after continuous interscalene brachial plexus blocks have also been reported [7]. Usually, these are related to phrenic nerve blockade and resultant hemidiaphragm paresis. It is believed that some amount of paralysis occurs in 100% of single-shot interscalene blocks, but only the continuous blocks seem to result in larger, symptomatic pleural effusions [7-8]. Here, it would seem that the single injection block performed under ultrasound guidance, is not likely to have caused the significant hydrothorax alone. Hemidiaphragm paresis may have contributed to the effusion, but it was unlikely the main etiology.

Considering the quick development of this hydrothorax, extravasated fluid in the chest cavity versus surgical disruption of the pleural space are the most likely causes. Several reports have described which common factors can be controlled to mitigate the risk of substantial fluid extravasation during shoulder arthroscopy (Table 1) [6]. 

**Table 1:** Factors associated with increased fluid extravasation during arthroscopic procedures.

Factors leading to increase fluid extravasation
Pump pressure > 150 mmHg
Large volume of irrigation
Lateral decubitus position
Obesity
Older age
Resection of glenohumeral capsule
Anatomic abnormalities leading to tears in the parascapular musculature

**Figure 5:**
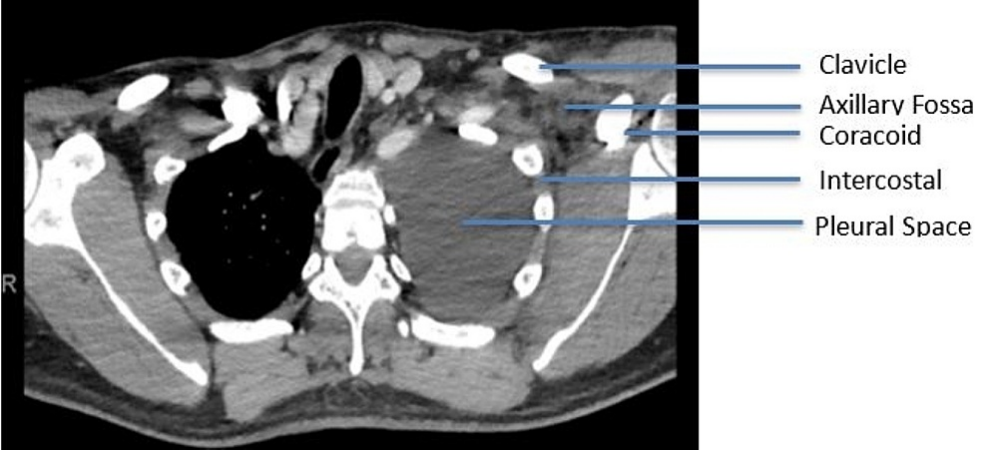
Postoperative axial slice from the CT chest revealing anatomic relationship of surgical site to pleural space Layers include coracoid process, axillary fossa, intercostal muscles, parietal pleura, and pleural space.

This patient was at a higher risk for pleural effusion from arthroscopic surgery due to the revisional nature, and anatomic location of this surgery. However, because of the large amount of fluid development in such a short period of time, there is a concern that there may have been some type of penetrating injury versus congenital communication to the pleura that resulted in direct communication of the surgical site and the pleural space (Figure 5). No repeat drilling was performed during the surgery so it seems unlikely that injury from the surgery itself caused this buildup. Due to the revisional nature of this surgery, there may have been some abnormal anatomy or prior injury that led to such significant hydrothorax, but the true cause or causes will never be known.

## Conclusions

Development of large pleural effusions after shoulder arthroscopy is a rare, but possibly life-threatening complication. The presentation of this case will demonstrate to future practitioners the need for a high index of suspicion for hydrothorax if their patients' respiratory status decompensate following shoulder arthroscopy. Additionally, patients that are at increased risk of developing a large postoperative pleural effusion should be monitored more closely. Such things to monitor would include fluid volume infusion compared to return, pump pressure, and total operative time. This may decrease potential complications including airway obstruction, tension hydrothorax, and airway edema. By at least considering this as a possibility, definitive management can be undertaken and, as long as it is promptly executed, can result in an excellent patient outcome and prevent potentially life-threatening situations.

## References

[1] Weber SC, Abrams JS, Nottage WM (2002). Complications associated with arthroscopic shoulder surgery. Arthroscopy.

[2] Chellam S, Chiplonkar S, Pathak K (2015). Change in neck circumference after shoulder arthroscopy: an observational study. Indian J Anaesth.

[3] Hynson JM, Tung A, Guevara JE, Katz JA, Glick JM, Shapiro WA (1993). Complete airway obstruction during arthroscopic shoulder surgery. Anesth Analg.

[4] Orebaugh SL (2003). Life-threatening airway edema resulting from prolonged shoulder arthroscopy. Anesthesiology.

[5] Ko SH, Jung KH, Cha JR, Song MC (2015). Severe airway obstruction and pleural effusion after arthroscopic shoulder surgery: a case report. Arthrosc Orthop Sport Med.

[6] Memon M, Kay J, Gholami A, Simunovic N, Ayeni OR (2018). Fluid extravasation in shoulder arthroscopic surgery: a systematic review. Orthop J Sports Med.

[7] Yang CW, Jung SM, Cho CK (2010). Pleural effusion and atelectasis during continuous interscalene brachial plexus block - a case report. Korean J Anesthesiol.

[8] Urmey WF, Talts KH, Sharrock NE (1991). One hundred percent incidence of hemidiaphragmatic paresis associated with interscalene brachial plexus anesthesia as diagnosed by ultrasonography. Anesth Analg.

